# Combination of Carbon Dioxide Angiography and Outback® Elite for Revascularization of a Patient with Renal Insufficiency with Bilateral Femoropopliteal Chronic Total Occlusions

**DOI:** 10.1155/2017/8632747

**Published:** 2017-07-09

**Authors:** Yuhei Nojima, Shinsuke Nanto, Hidenori Adachi, Madoka Ihara, Tetsuya Kurimoto

**Affiliations:** Department of Cardiology, Nishinomiya Municipal Central Hospital, Nishinomiya, Japan

## Abstract

A new reentry device (Outback Elite) system has been available in Japan since June 2016. This new device enables easier treatment of chronic total occlusion (CTO) in the lower extremities. We report a case of a woman in her 70s who underwent revascularization using this new device twice to treat both of her femoropopliteal CTO lesions. She was referred to our hospital complaining of intermittent claudication in both legs. She had a long history of diabetes mellitus complicated with severe chronic kidney disease. Her estimated glomerular filtration rate was <20. She refused surgical revascularization; therefore, we performed our treatment without iodine contrast medium. First, magnetic resonance imaging was performed to confirm that the CTO lesions had caused severe claudication before intervention. Subsequently, the Outback Elite device and carbon dioxide (CO_2_) angiography made it possible to revascularize both of her legs without iodine contrast medium. At 6 months after the procedures, we did not observe exacerbation of claudication in her legs.

## 1. Introduction

Iodine contrast medium is considered as the gold standard for endovascular treatment (EVT) because it provides much information needed to select a treatment strategy. However, the problem of contrast-induced nephropathy (CIN) remains associated with EVT. In high-risk patients, such as those with chronic renal impairment, diabetes mellitus, and congestive heart failure and the elderly, the incidence of CIN is >20%–30% [1]. Carbon dioxide (CO_2_) angiography is an alternative method that has been used since the 1970s for patients with renal impairment or hypersensitivity to iodine [2]. Subintimal angioplasty (SIA) with recanalization of SFA-CTO is an acceptable first-line therapy that was first described by Bolia et al. [3]. However, the reentry procedure often becomes difficult when the wire crosses the distal true lumen after excessive reach beyond the subintimal space. The Outback Elite is a new device that can lead to successful outcomes of revascularization in SFA-CTO. Here we report a patient with chronic renal impairment who underwent revascularization using the reentry device with CO_2_ angiography guidance in bilateral SFA-CTO.

## 2. Case Report

**Figure 1:**
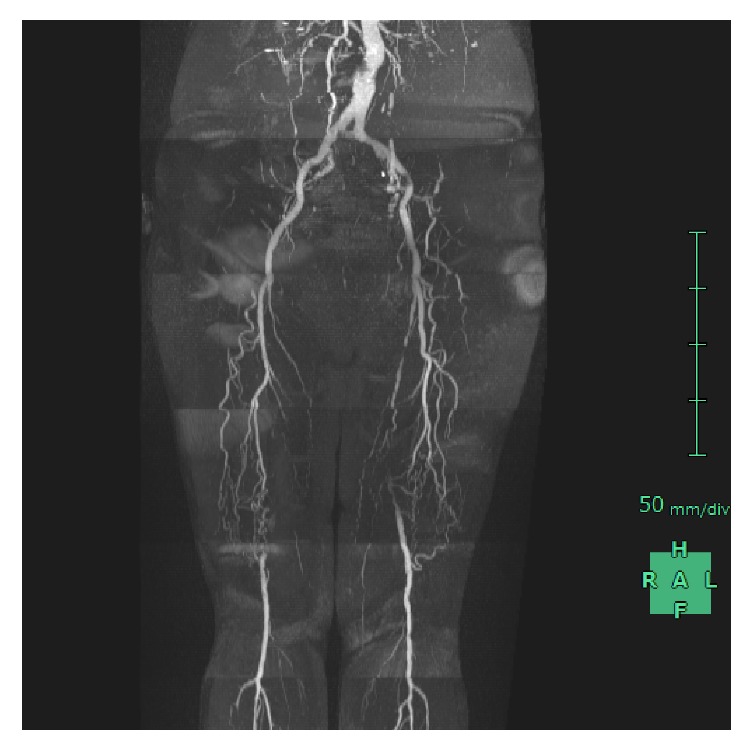
MRI showed that both of the patient's SFAs were already occluded from bifurcation of the deep femoral artery. MRI: magnet resonance imaging; SFA: superficial femoral artery.

A 76-year-old woman with a long history of diabetes mellitus was referred to our hospital with complaints of severe claudication in bilateral lower limbs. She had noticed claudication symptoms approximately 10 years earlier. Recently, she could not walk 100 m without needing to rest. Her long history of diabetes mellitus required that she take multiple oral medications but not insulin. Unfortunately, her condition was complicated by diabetic nephropathy. Laboratory studies revealed increased HbA1c level [6.7% (NGSP); reference value (RV) 4.6%–6.2%], blood urea nitrogen (45.3 mg/dL; RV 7–22 mg/dL), creatinine (2.07 mg/dL; RV 0.4–0.8 mg/dL), and a decreased estimated glomerular filtration rate of 19.16. The ankle-brachial pressure index (ABI) was 0.72 on the right side and 0.74 on the left side. We gave her the option of one of two revascularization methods: femoropopliteal bypass surgery or EVT. Both treatments have benefits and disadvantages for patients. After careful consideration, she decided to undergo revascularization by EVT. Before EVT, we needed more information about the status of her arteries in both legs; therefore, we performed magnetic resonance imaging (MRI). The MRI results confirmed that both of her SFAs were occluded from bifurcation of the deep femoral artery (Figure 1).

### 2.1. Revascularization of the Left SFA-CTO (Figure 2)

Initially, we performed EVT for the left SFA-CTO. A 6 Fr Destination® catheter (Terumo Corporation, Tokyo, Japan) was inserted from the right femoral artery into the left common femoral artery as a contralateral antegrade wiring approach. A 0.018-inch Treasure® guidewire (Asahi Intecc Co., Ltd., Nagoya, Japan) with a NaviCross® microcatheter (Terumo Corporation, Tokyo, Japan) was able to penetrate the proximal fibrous cap of the CTO. After advancing the microcatheter into the CTO site a little, we exchanged the 0.018-inch wire for a hydrophilic 0.035-inch, 1.5-mm, J-type, shaped-tip wire. A knuckle-shaped hydrophilic 0.035-inch wire was used to easily cross the middle of the CTO site and to eventually capture the distal site of the CTO. The 0.035-inch wire was not in the true lumen, however, so we subsequently used a reentry device called an Outback Elite catheter (Cordis, Florida, USA) to attempt to gain access to the true lumen. The device was delivered to the distal subintimal space adjacent to the reconstructed area of the distal true lumen, and some effort was needed to successfully penetrate using a hollow needle. Two orthogonal angiographic views were taken to confirm the correct direction: an L-shaped fluoroscopic marker enabled orientation of the tip toward the reentry site and, to attain the appropriate position, we needed to fine-tune the positioning at the site and used a T-shaped fluoroscopic marker to confirm the desired alignment. Next, a 22 G nitinol reentry cannula was plunged into the distal true lumen in the distal SFA. A 0.014-inch Chevalier Floppy® extrasupport wire (Cordis, Florida, USA) was advanced into the true lumen. Then, the reentry device was removed, and we used a SABER X® 5 × 60 mm (Cordis, Florida, USA) to perform balloon dilatation in the CTO lesion. Next, two 6.0 × 100 mm SMART® stents (Cordis, Florida, USA) were implanted from the proximal to middle portion of the left SFA. We performed postballoon dilatation using the same balloon. All procedures were performed without iodine contrast medium. The patient's left ABI improved from 0.74 to 0.98.

### 2.2. Revascularization of the Right SFA-CTO (Figure 3)

One month after the procedure for the left SFA-CTO, we performed EVT for the right SFA-CTO. A 6 Fr Parent Plus60™ sheath was inserted from the right femoral artery as an ipsilateral antegrade wiring approach. A 0.018-inch Treasure guidewire with a 4.0 Fr CXI™ support catheter (Cook Medical, Bloomington, IN, USA) was used to penetrate the fibrous cap of the CTO. Advancing the microcatheter into the CTO site, we exchanged the 0.018-inch wire for a hydrophilic 0.035-inch, 1.5-mm, J-type, shaped-tip wire. A knuckle-shaped hydrophilic 0.035-inch wire was used to reach the distal site of the CTO. The 0.035-inch wire was intended to be placed in the false lumen, but subsequent attempts to gain access to the true lumen were performed using an Outback Elite catheter. We performed CO_2_ angiography to guide exact positioning of the needle and stents. A 0.014-inch extrasupport wire was advanced into the true lumen. Then, the reentry device was removed, and we performed balloon dilatation using a 5 × 60 mm balloon in the CTO lesion. Subsequently, two SMART stents (7.0 × 100 mm and 6.0 × 150 mm) were implanted from the proximal to middle portion of the right SFA. We performed postballoon dilatation using the same balloon. All procedures were performed without iodine contrast medium. Subsequent examinations showed that the patient's left ABI improved from 0.72 to 0.91.

## 3. Discussion

Generally speaking, expansion of a subintimal stent is less than that of an intraplaque stent and contributes to long-term outcomes, particularly in coronary arteries. For treating CTO, intraplaque angioplasty (IPA) is preferable over SIA to maintain chronic patency for CTO lesions. However, some studies have found no statistically significant differences between IPA and SIA for treating SFA-CTO [4–6]. Therefore, we have frequently adopted SIA using a 0.035-inch knuckled-wire technique. This technique is feasible and easy to perform except for reentering the distal true lumen at the CTO site [4, 5]. However, the guidewire may not pass the subintimal lumen in all cases. Otherwise, the intraluminal procedure using a 0.014- or 0.018-inch guide wire does not always succeed in capturing the distal true lumen. If the distal true lumen cannot be captured, retrograde access may be a useful and feasible option [7]. However, the retrograde technique requires a certain amount of experience with EVT. Thus, a reentry device was developed to overcome procedural failures and improve success rates after subintimal passage through the CTO. Recent studies have reported >95% procedural success rates in achieving revascularization using the Outback catheter [8]. In contrast, Shin et al. found that significant calcification at the reentry site was a strong predictor of procedural failure after use of this reentry device [9]. Our patient underwent EVT for CTOs in both SFAs, and these revascularizations intentionally needed the reentry device in both of her legs. The first session used the contralateral approach, and the second session used the ipsilateral approach. On the basis of our experience, if using a reentry device, the ipsilateral approach would be preferred to the contralateral approach. The contralateral approach makes it more difficult for a reentry device to cross over the aortic bifurcation; therefore, in this approach, previous dilatation of the subintimal lumen is required to insert such a bulky device. In our 2 experiences, use of a reentry device required more time in the contralateral approach than in the ipsilateral approach. Therefore, we recommend using and/or being prepared to use the ipsilateral approach with a 6 Fr system as a standby if this new reentry device is used. Lastly, operators should be aware of characteristics of CO_2_ angiography. CO_2_ angiography is particularly advantageous for patients at risk of CIN and allergic reactions against iodine contrast medium (ICM) [2]. Moreover, we consider CO_2_ as effective as ICM within the femoropopliteal lesions [10]. Complications following the CO_2_ angiography have been reported, including vapor-lock, which occurs upon entrapment of CO_2_ at the origin of a vessel, thereby impairing flow and potentially resulting in bowel ischemia especially in EVT for aortoiliac or renal lesions [11].

## 4. Conclusions

To the best of our knowledge, this is the first report to use an Outback Elite catheter under CO_2_ angiography. Six months after the procedure, we did not observe exacerbation of claudication in our patient's legs. We found that CO_2_ angiography for treating SFA-CTO lesions was useful and effective. EVT for SFA-CTO using SIA or IPA would be expected to give the same results. This combination therapeutic technique could potentially lead to a new intervention era in which contrast dye is not needed.

## Figures and Tables

**Figure 2:**
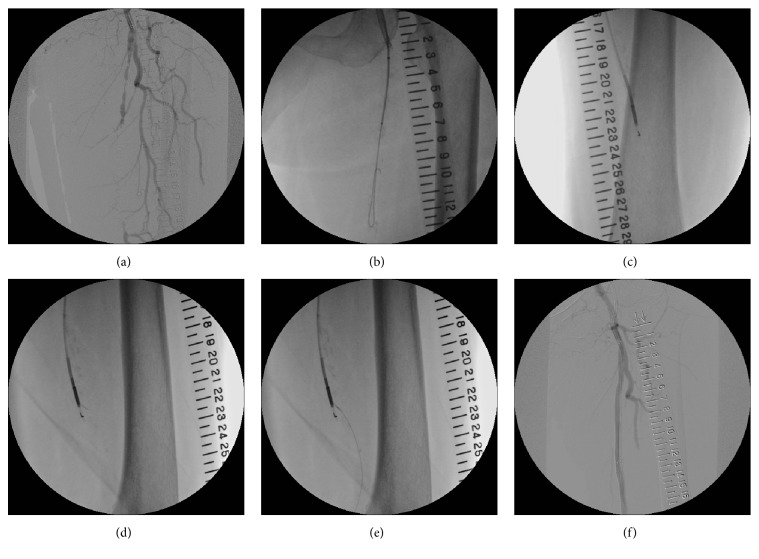
Initially, we performed EVT to treat the occlusion of her left SFA. We used a new reentry device called an Outback Elite and the contralateral approach. Two orthogonal angiographic views are essential to confirm the right direction: an L-shaped fluoroscopic marker provides the orientation of the tip toward the reentry site, and fine tuning of the positioning is needed when using a T-shaped fluoroscopic marker to confirm the desired alignment. Next, a 22 G nitinol reentry cannula was plunged into the distal true lumen in the distal SFA. A 0.014-inch extrasupport wire was advanced into the true lumen. EVT: endovascular therapy; SFA: superficial femoral artery.

**Figure 3:**
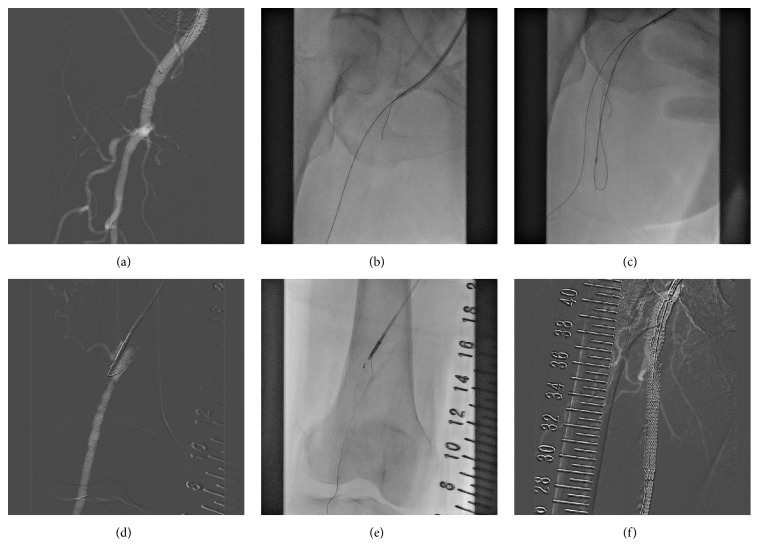
We subsequently performed EVT to treat the occlusion of the right SFA. We used an Outback Elite and the ipsilateral approach. This new reentry device enabled successful performance of EVT for this tough CTO lesion. The ipsilateral approach is better than the contralateral approach to bring the reentry device into the subintimal space. EVT: endovascular therapy; SFA: superficial femoral artery; CTO: chronic total occlusion.
